# Pulse Generation in the Quorum Machinery of *Pseudomonas aeruginosa*

**DOI:** 10.1007/s11538-017-0288-z

**Published:** 2017-05-19

**Authors:** Cicik Alfiniyah, Martin A. Bees, A. Jamie Wood

**Affiliations:** 1 Department of Mathematics, University of York, York, YO10 5DD UK; 2 Department of Mathematics, Universitas Airlangga, Surabaya, 60115 Indonesia; 3 Department of Biology, University of York, York, YO10 5DD UK

**Keywords:** *Pseudomonas aeruginosa*, Quorum sensing, Excitable behaviour, Bifurcation analysis

## Abstract

*Pseudomonas aeruginosa* is a Gram-negative bacterium that is responsible for a wide range of infections in humans. Colonies employ quorum sensing (QS) to coordinate gene expression, including for virulence factors, swarming motility and complex social traits. The QS signalling system of *P. aeruginosa* is known to involve multiple control components, notably the *las*, *rhl* and *pqs* systems. In this paper, we examine the *las* system and, in particular, the repressive interaction of *rsaL*, an embedded small regulative protein, employing recent biochemical information to aid model construction. Using analytic methods, we show how this feature can give rise to excitable pulse generation in this subsystem with important downstream consequences for rhamnolipid production. We adopt a symmetric competitive inhibition to capture the binding in the lasI–rsaL intergenic region and show our results are not dependent on the exact choice of this functional form. Furthermore, we examine the coupling of *lasR* to the *rhl* system, the impact of the predicted capacity for pulse generation and the biophysical consequences of this behaviour. We hypothesize that the interaction between the *las* and *rhl* systems may provide a quorum memory to enable cells to trigger rhamnolipid production only when they are at the edge of an established aggregation.

## Introduction

*Pseudomonas aeruginosa* is a common Gram-negative bacterium responsible for a wide range of infections, including those of the urinary and gastrointestinal tract, the skin, and, most prominently, the respiratory system in immunocompromised hosts and sufferers of cystic fibrosis (CF). *P. aeruginosa* is a well-studied opportunistic pathogen in many contexts; it is well known for its ability to form biofilms (O’Loughlin et al. 2013; Singh et al. 2015), its swarming behaviour (Daniels et al. 2004; Shrout et al. 2006), its rapid acquisition of resistance to antibiotics (Shih and Huang 2002) and its quorum sensing (QS) behaviour (Fuqua et al. 2001). QS in *P. aeruginosa* is of particular interest because the mechanism is more complex than the originally discovered, prototypical Lux homolog positive-feedback loop (e.g. James et al. 2000; Shadel and Baldwin 1991) and the number of genes regulated by QS is large (Sitnikov et al. 1995), especially those associated with virulence (O’Loughlin et al. 2013). Mathematical models of QS in *Pseudomonas aeruginosa* have received a lot of attention. They provide the formalism to summarize current understanding as well as the means to explore mechanisms and evaluate emergent solution behaviour. Here, we develop a model description, employing recent genomic information and bioinformatic techniques, and explore mechanisms for the generation of pulses and memory effects for downstream rhamnolipid production.

**Fig. 1:**
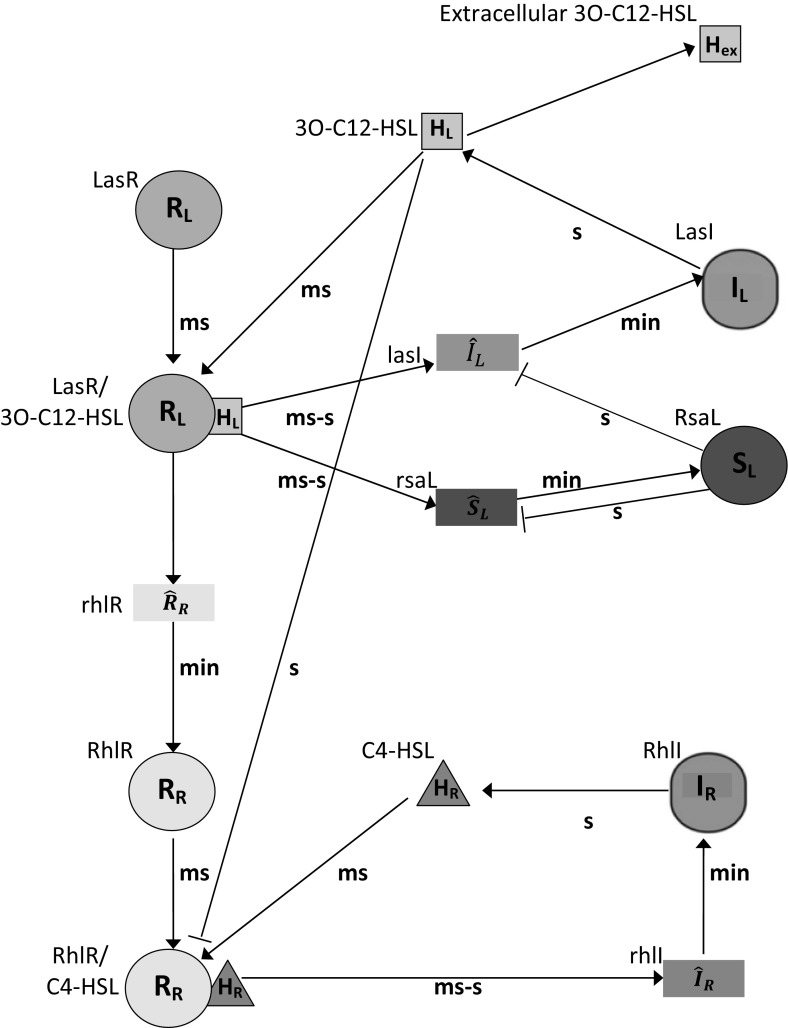
The quorum sensing signalling system in *Pseudomonas aeruginosa* is composed of *las* and *rhl* systems. *Arrows* and *barred arrows* indicate activating (positive) and inhibiting (negative) regulatory interactions, respectively. *Shapes* on the diagram depict autoregulation terminology. *Letters* associated with *each arrow* reflect the associated time scale (ms = millisecond, s = second, and min = minute). *Symbols* associated with each *shape* are detailed in Table 1. Adopted from Van Delden and Iglewski (1998) (Color figure online)

In *P. aeruginosa* quorum sensing is governed by a hierarchical Luxl/LuxR system, which consists of two homolog pairs: LasI/LasR and RhlI/RhlR (Miller and Bassler 2001). Under this process, formation of the HSL autoinducers *N*-(3-oxododecanoyl)-HSL and *N*-(butyryl)-HSL is synthesised by LasI and RhlI, respectively (see Fig. 1). It should be noted, however, that signalling systems of *las* and *rhl* are specific in their activation of autoinducers, i.e. *N*-(3-oxododecanoyl)-HSL is unable to activate RhlR and, similarly, LasR cannot be activated by *N*-(butyryl)- HSL (Latifi et al. 1995; Pearson et al. 1997). Although biochemically independent, the *las* system is able to exert control of the *rhl* system through the transcriptional promotion of the RhlR gene by LasR/*N*-(3-oxododecanoyl)-HSL (Pesci et al. 1997; Latifi et al. 1996). As well as gene regulation effects, the *rhl* system has an important function of modulating rhamnolipid production via *rhlAB*. Rhamnolipids are particularly important in swarming motility where they are postulated to lower surface tension and allow expansion of the colony through their surfactant and wetting properties, driving the bacteria to swarm on surfaces (Glick et al. 2010; Kohler et al. 2000). In addition, a quinolone system (Dubern and Diggle 2008) may also modulate these interconnecting feedback loops (for simplicity, we do not model this aspect of QS here).

**Table 1:** Description of dimensional variables

Variable	Description	Unit
$R_{L}$	LasR	nM
$H_{L}$	3O-C12-HSL	nM
$R_{LH}$	LasR/3O-C12-HSL complex	nM
$I_{L}$	LasI	nM
$\hat{I}_{L}$	lasI mRNA	nM
$S_{L}$	RsaL	nM
$\hat{S}_{L}$	rsaL mRNA	nM
$R_{R}$	RhlR	nM
$H_{R}$	C4-HSL	nM
$R_{RH}$	RhlR/C4-HSL complex	nM
$\hat{R}_{R}$	rhlR mRNA	nM
$I_{R}$	RhlI	nM
$\hat{I}_{R}$	rhlI mRNA	nM

The first models of QS in *P. aeruginosa* were of the Lux (James et al. 2000) and the Las systems (Dockery and Keener 2001). Both descriptions, and subsequent models, highlight the existence of a fold bifurcation structure for the concentration of the response regulator in response to bulk cell concentration. The seminal paper by Dockery and Keener (2001) provides the foundation for the emergence of QS based on formal mass action arguments. However, there have been significant increases in biochemical knowledge of this system in the last 15 years. Fagerlind et al. (2003) constructed a large mass action model of the coupled Las and Rhl systems, including the effects of both RsaL and Vfr. This work also emphasizes the existence of the classic twofold bifurcation diagram for the activation level of the system (typically for the levels of liganded LasR) with respect to the external concentration of HSL. Subsequent work Fagerlind et al. (2005) then explored how anti-virulence drugs (followed by Skindersoe et al. 2008) are able to quorum quench this system. The qualitative model of Viretta and Fussenegger (2004) does not include the effect of the *rsaL* negative loop and lacks the capacity to deal with the nonlinear effects predicted here. The production of rhamnolipid was modelled and tested empirically by Chen et al. (2004). The exhaustive rule-based approach of Schaadt et al. (2013) includes the effect of *rsaL* but does not include the kinetic possibilities that emerge from nonlinear interactions. There are many other studies on this system but, increasingly, these are based on the perspective of computational rather than mathematical modelling (e.g. Dockery and Keener 2001; Fagerlind et al. 2005; Schaadt et al. 2013), typically with a non-mechanistic emphasis.

In this article, we focus in detail on the Las system and its internal regulation and modulation for individual cell. The *las* system is composed of LasI, autoinducer *N*-(3-oxododecanoyl)-HSL, LasR and RsaL (Pesci et al. 1997). Importantly, biochemical evidence has now firmly established that LasR exists as a dimer in solution, with each monomer liganded by a single HSL (*N*-(3-oxododecanoyl)-HSL) molecule with additional evidence supporting higher multimers upon DNA binding (Schuster and Greenberg 2006). This is in contrast to assumptions of Dockery and Keener (2001) who assume a simpler mechanism for binding. Mathematically, the biochemical evidence is consistent with a Hill number of at least two and possibly much higher. In contrast, the RsaL transcriptional repressor is a helix-turn-helix protein that binds the promoter of *lasI* (De Kievit et al. 2002) and exists as a monomer in the cell (Rampioni et al. 2007a), leading to a Hill number of one. The transcription of both genes is promoted and regulated via binding of the two proteins to the same intergenic region between the lasI and rsaL operons, so except for rates the functional form for the transcription is likely to be identical (but we discuss variations of this in “Appendix 1”). This improvement in biochemical knowledge offers clear guidance for expected mathematical forms in the equations.

We begin with an investigation of the behaviour of gene regulation by constructing a mathematical model of a single cell. In order to focus on a biologically plausible region of parameter space, we conduct an extensive search of published data, noting deviations from modelling choices in the literature. The dynamical system exhibits a range of interesting solution behaviour. We find that bistable solutions and oscillations are possible and explore the bifurcation structure. Of significant interest is the potential for excitability in the system, in the presence of both single and multiple steady states, such that a modest perturbation from a low-level steady state triggers a large-amplitude excursion around phase space that eventually returns the system to low steady state. The extracellular concentration of HSL can have a significant impact on the dynamics. As signal molecules can accumulate and diffuse in the external environment, we demonstrate how pulse generation of the *las* system can be propagated in space.

This paper is organized as follows. In Sect. 2, we describe in greater detail the biological system as well as the mathematical approach. In Sect. 3, we conduct a numerical exploration of our model, highlighting the key parameters in determining LasI and RsaL equilibria. We then employ extracellular concentration as a driving factor in the dynamical behaviour of the system. We conclude by discussing our findings, identify challenges and suggest future work in Sect. 4.

## Methods

We construct the governing equations using mass action kinetics, except where noted, guided by the literature (e.g. Fagerlind et al. 2003). First, consider the LasR regulator ($R_{L}$), its binding activator, the autoinducer 3O-C12-HSL $(H_{L})$, and the complex LasR/3O-C12-HSL $(R_{LH})$. If LasR associates at rate $k_{L}^{+},$ dissociates at rate $k_{L}^{-},$ is produced at a rate $\beta _0$ and degrades or dilutes at rate $\gamma _{L},$ then we may write

1 $$\begin{aligned} \frac{\mathrm{d}R_{L}}{\mathrm{d}t}=-k_{L}^{+}R_{L}H_{L}+k_{L}^{-}R_{LH} + \beta _0 -\gamma _{{L}}R_{L}, \end{aligned}$$

In a similar manner, we formulate an equation for the complex, with degradation or dilution rate $\gamma _{RL}$, such that

2 $$\begin{aligned} \frac{\mathrm{d}R_{LH}}{\mathrm{d}t}=k_{L}^{+}R_{L}H_{L}-k_{L}^{-}R_{LH}-\gamma _{RL}R_{LH}. \end{aligned}$$

where $\beta _0, \gamma _{L} , \gamma _{RL}$ are positive constants.

New biochemical data have indicated approximately steady cellular levels (Ishihama et al. 2014) of many transcription factors and, therefore, we hypothesize it is the change in activation rather than production per se that dictates the dominant dynamics; unlike previous studies that consider the regulated production of LasR. Therefore, we shall assume the association and dissociation processes occur over a sufficiently fast time scale that the production and loss terms can be safely neglected: we disregard terms multiplied by $\beta _0$, $\gamma _{L}$ and $\gamma _{RL}$.

The autoinducer 3O-C12-HSL $(H_{L})$ is created in the system via the activity of the LasI synthase $(I_{L})$, which we take to be at rate $\beta _{HL}$, and is naturally lost from the system at rate $\gamma _{HL}$. The most significant loss of the autoinducer from the cell is via diffusion through the cell membrane, a process we account for separately. Taking a simplified description of diffusion we can express the diffusive term as being proportional to the concentration difference across the membrane of $H_{L}$, where we take the extracellular concentration to be $H_\mathrm{ex}$. Therefore, $D_{HL}$ represents an additional loss rate, which is multiplied by the concentration difference, $H_{L}-H_\mathrm{ex},$ yielding

3 $$\begin{aligned} \frac{\mathrm{d}H_{L}}{\mathrm{d}t}=\beta _{HL}I_{L}-\gamma _{HL}H_{L}-D_{HL}(H_{L}-H_\mathrm{ex}). \end{aligned}$$

The enzyme LasI $(I_{L})$ is produced by the lasI gene through a transcription and translation process of lasI mRNA $(\hat{I}_{L})$ at rate $\alpha _{L}$ and degrades at rate $\gamma _{IL},$ such that

4 $$\begin{aligned} \frac{\mathrm{d}I_{L}}{\mathrm{d}t}=\alpha _{L}\hat{I}_{L}-\gamma _{IL}I_{L}. \end{aligned}$$

In a similar fashion, the inhibitor RsaL $(S_{L})$ is produced by rsaL genes through transcription and translation of rsaL mRNA $(\hat{S}_{L})$ at rate $\alpha _{S}$ and degrades at rate $\gamma _{S},$ providing

5 $$\begin{aligned} \frac{\mathrm{d}S_{L}}{\mathrm{d}t}=\alpha _{S}\hat{S}_{L}-\gamma _{S}S_{L}. \end{aligned}$$

Transcription at the lasI promoter site $(\hat{I}_{L})$ is activated by the LasR/3O-C12-HSL complex $(R_{LH})$. The production process is assumed to follow a Hill form with a Hill number *p*. Recent biochemical evidence strongly suggests a Hill number $>1$ in contrast to the arguments of Dockery and Keener (2001); the activated form of lasR is at least dimeric (Schuster and Greenberg 2006), and it is possible that it forms a tetramer on the DNA. For mathematical simplicity, we adopt the smaller potential value, $p=2$, in the analysis that follows. In addition, the RsaL transcriptional repressor has been shown to bind to the lasI-rsaL intergenic region but, unlike LasR, RsaL is a helix-turn-helix protein that exists as a monomer in the cell (Rampioni et al. 2007a), and thus we will adopt Hill number $q=1$ for the inhibition factor for production of RsaL $(\hat{S}_{L})$, mathematically equivalent to a Michaelis–Menten equation. The transcription of both genes is promoted and regulated via binding of the two proteins to the same intergenic region between the lasI and rsaL operons, so the functional form for the transcription is identical, with the exception of the numerical values of the transcription and loss rates. Note that this implies a negative feedback relation between the RsaL protein and its own production, which has not been historically represented on graphical depictions of the *las* system, but strongly implied by analysis of Rampioni et al. (2007a). Biochemically there is insufficient evidence to determine whether the binding in the intergenic region results in competitive, uncompetitive or non-competitive binding system, or indeed whether there is a symmetry in the expression rates in each direction with all configurations of binding at the intergenic region. In the analysis below, we adopt a symmetric competitive binding form (we choose $K_L=K_S$ in the analysis below), but show in “Appendix 1” that qualitatively the results are not dependent on this functional choice. With basal expression of $\beta _{L0}$ and a loss rate of $\gamma _{mL}$, this leads to the following expression for lasI:

6 $$\begin{aligned} \frac{\mathrm{d}\hat{I}_{L}}{\mathrm{d}t}=\beta _{L}\frac{R_{LH}^{p}}{K_{L}^{p}(1+S^q_{L}/K^q_{SL})^{p}+R_{LH}^{p}}-\gamma _{mL}\hat{I}_{L}+\beta _{L0}. \end{aligned}$$

Similarly, with basal expression of $\beta _{S0}$ and a loss rate of $\gamma _{S}$ for rsaL we obtain

7 $$\begin{aligned} \frac{\mathrm{d}\hat{S}_{L}}{\mathrm{d}t}=\beta _{S}\frac{R_{LH}^{p}}{K_{S}^{p}(1+S^q_{L}/K^q_{SL})^{p}+R_{LH}^{p}}-\gamma _{mS}\hat{S}_{L}+\beta _{S0}. \end{aligned}$$

The numerical values of the temporal processes are such that we are able to make a number of assumptions regarding the timescales to simplify the system. We assume that the dominant, slowest processes are protein production from mRNA via translation and folding. Therefore, other processes, namely the liganding of regulators, DNA binding, synthetase operation, and the transcription of DNA, are much faster and we can assume that four of our differential equations are at a quasi-steady state, such that

8 $$\begin{aligned} \hat{I}_{L}=\frac{\beta _{L}R_{LH}^{p}}{\gamma _{mL}\left( K_{L}^{p}\left( 1+\frac{S^q_{L}}{K^q_{SL}}\right) ^{p}+R_{LH}^{p}\right) }+\frac{\beta _{L0}}{\gamma _{mL}}, \end{aligned}$$

and

9 $$\begin{aligned} \hat{S}_{L}=\frac{\beta _{S}R_{LH}^{q}}{\gamma _{mS}\left( K_{S}^{p}\left( 1+\frac{S^q_{L}}{K^q_{SL}}\right) ^{p}+R_{LH}^{p}\right) }+\frac{\beta _{L0}}{\gamma _{mS}}. \end{aligned}$$

From Eqs. (1) and (2), we have a *Moiety conservation* equation

10 $$\begin{aligned} \frac{\mathrm{d}R_{LH}}{\mathrm{d}t}+\frac{\mathrm{d}R_{L}}{\mathrm{d}t}=0 \Rightarrow R_{LH}+R_{L}=R_{L0}, ~\mathrm{where }~R_{L0}\,\mathrm{is~a~ constant.} \end{aligned}$$

Initially, we make the simplifying assumption that HSL diffusion is rapid and, therefore, that the equation for HSL, $H_{L}$ can also be written in a quasi-steady state, providing

11 $$\begin{aligned} H_{L}=\frac{\beta _{HL}I_{L}}{D_{HL}}+H_\mathrm{ex}. \end{aligned}$$

Later, we relax this constraint. With the simplifications above, the system of equations for the * las *system, constructed for the LasI and RsaL loops, becomes just two differential equations. By assuming there is negligible basal production of lasI and rsal genes $(\beta _{L0}=0$ and $\beta _{S0}=0$), the governing equations become

12 $$\begin{aligned} \frac{\mathrm{d}I_{L}}{\mathrm{d}t}=\frac{\alpha _{L}\beta _{L}}{\gamma _{mL}}\frac{R_{L0}^{2}H_{L}^{2}}{K_{L}^{2}\left( 1+\frac{S_{L}}{K_{SL}}\right) ^{2}\left( H_{L}+\frac{k_{L}^{-}}{k_{L}^{+}}\right) ^{2}+R_{L0}^{2}H_{L}^{2}}-\gamma _{IL}I_{L}, \end{aligned}$$

and

13 $$\begin{aligned} \frac{\mathrm{d}S_{L}}{\mathrm{d}t}=\frac{\alpha _{S}\beta _{S}}{\gamma _{mS}}\frac{R_{L0}^{2}H_{L}^{2}}{K_{S}^{2}\left( 1+\frac{S_{L}}{K_{SL}}\right) ^{2}\left( H_{L}+\frac{k_{L}^{-}}{k_{L}^{+}}\right) ^{2}+R_{L0}^{2}H_{L}^{2}}-\gamma _{S}S_{L}, \end{aligned}$$

with $H_{L}$ as in Eq. (11).

We nondimensionalize this model by writing

14 $$\begin{aligned} \eta =\frac{\beta _{HL}k_{L}^{+}}{D_{HL}k_{L}^{-}}I_{L}, \xi =\frac{S_{L}}{K_{SL}}, \mathrm{and}\quad \tau =\frac{\alpha _{L}\beta _{L}\beta _{HL}k_{L}^{+}}{ \gamma _{mL}D_{HL}k_{L}^{-}}t, \end{aligned}$$

so that (12) and (13) become

15 $$\begin{aligned} \frac{\mathrm{d}\eta }{\mathrm{d}\tau }= & {} \frac{\left( \eta +a_{1}\right) ^{2}}{a_{2}\left( 1+\xi \right) ^{2}\left( \eta +a_{1}+1\right) ^{2}+\left( \eta +a_{1}\right) ^{2}}-a_{3}\eta ,\end{aligned}$$

16 $$\begin{aligned} \frac{\mathrm{d}\xi }{\mathrm{d}\tau }= & {} \frac{a_{4}\left( \eta +a_{1}\right) ^{2}}{a_{2}\left( 1+\xi \right) ^{2}\left( \eta +a_{1}+1\right) ^{2}+\left( \eta +a_{1}\right) ^{2}}-a_{5}\xi , \end{aligned}$$

where

17 $$\begin{aligned}{}\begin{array}[t]{cccc} a_{1}=\frac{H_\mathrm{ex}k_{L}^{+}}{k_{L}^{-}}, &{} &{} &{} a_{2}=\left( \frac{K_{L}}{R_{L0}}\right) ^{2}, \\ &{} &{} &{} \\ a_{3}=\frac{\gamma _{IL}\gamma _{mL}D_{HL}k_{L}^{-}}{\alpha _{L}\beta _{L}\beta _{HL}k_{L}^{+}}, &{} &{} &{} a_{4}=\frac{\alpha _{S}\beta _{S}}{\gamma _{IL}\gamma _{mS}K_{SL}}a_{3}, \\ &{} &{} &{} \\ a_{5}=\frac{\gamma _{S}}{\gamma _{IL}}a_{3}. \end{array} \end{aligned}$$

Here, $a_{1}$, $a_{2}$, $a_{3}$, $a_{4}$, and $a_{5}$ are positive constants. The biological interpretation of this model is that $\xi$ inhibits the expression of both $\eta$ and $\xi$, which describes negative feedback from competitive inhibition by RsaL to the expression of both *lasI* and *rsaL* genes. The binding of RsaL to the bidirectional *lasI-rsaL* counters positive feedback, thereby balancing levels of HSL. In addition, both $\eta$ and $\xi$ degrade exponentially. The model thus constructed is a type of incoherent feed forward motif (Alon 2006) as can be seen from Fig. 1.

## Results

The non-dimensional set of differential Eqs. (15) and (16) have been investigated analytically with the assistance of Mathematica (10; Wolfram) and solved numerically using MATLAB (R2016a; MathWorks). We note that the fixed points of this system lie upon the straight line $a_{3} a_{4} \eta = a_{5} \zeta$ but substitution of this expression into either nullcline leads to a quintic equation for the fixed points that does not provide sufficient further simplification to yield general expressions for stability bounds.

### Parameter Ranges in the System

Table 2 lists parameters that have either been adopted from the literature based on experimental evidence or estimated, as stated. Moreover, some parameters are chosen for the following reasons. The basal production rate of genes-mRNA can be considered as similar to the basal transcription rate of a protein. This is because the transcriptional regulator protein activates genes-mRNA in a very fast process before encoding the protein. We take typical values of total concentration of LasR and LasR/3O-C12-HSL to be 200 nM, as for the concentration of QseB in *E. coli* (Ishihama et al. 2014). As described in the previous section, we simplify the governing equations by assuming quasi-steady states for the fast reactions. This results in just 5 non-dimensional parameters (see Table 3).

The dimensionless variable $a_{1}$ is proportional to $H_\mathrm{ex}$, the extracellular HSL concentration, an important factor in controlling both the intracellular HSL production and cell–cell communication. We shall see that $a_{1}$ influences the location of the key bifurcation.

**Table 2:** Parameters employed in the model

Par	Description	Standard value	Unit	Value/range	Comments (based on)/Ref
$k_{L}^{+}$	Rate of binding reaction between LasR and 3O-C12-HSL		nM$^{-1}$ min$^{-1}$		Ratio $\frac{k_{L}^{-}}{k_{L}^{+}}\sim 1000-2000$ (nM)
$k_{L}^{-}$	Dissociation reaction rate of LasR/3O-C12-HSL		min$^{-1}$		Ratio $\frac{k_{L}^{-}}{k_{L}^{+}}\sim 1000-2000$ (nM), Welch et al. (2000)
$\alpha _{L}$	Rate at which LasI produced by lasI mRNA	0.5	min$^{-1}$	0.5	2 min to translate protein, Alon (2006)
$\alpha _{S}$	Rate at which RsaL produced by rsaL mRNA	0.5	min$^{-1}$	0.5	2 min to translate protein, Alon (2006)
$\beta _{HL}$	Rate at which 3O-C12-HSL produced by LasI	$8\times 10^{2}$	min$^{-1}$	$8\times 10^{2}$	Raychaudhuri et al. (2005)
$\beta _{L}$	Max. production rate of LasI when lasI mRNA is activated by LasR/3O-C12-HSL	1	nM min$^{-1}$	1	Estimate
$\beta _{S}$	Max. production rate of RsaL when rsaL mRNA is activated by LasR/3O-C12-HSL	1	nM min$^{-1}$	1	Estimate
$\beta _{L0}$	Basal production rate of lasI mRNA	0.1	nM min$^{-1}$	0.1	Basal transcription rate of a protein, Alon (2006)
$\beta _{S0}$	Basal production rate of rsaL mRNA	0.1	nM min$^{-1}$	0.1	Basal transcription rate of a protein, Alon (2006)
$K_{L}$	Affinity constant between LasR/3O-C12-HSL and lasI mRNA	116	nM	1–1000	Alon (2006)
$K_{S}$	Affinity constant between LasR/3O-C12-HSL and rsaL mRNA	116	nM	1–1000	Alon (2006)
$K_{SL}$	Dissociation constant of inhibitor RsaL to lasI mRNA	185	nM	1–1000	Alon (2006)
$\gamma _{L}$	Degradation rate of LasR	0.01	min$^{-1}$	0.01	Alon (2006)
$\gamma _{IL}$	Degradation rate of LasI	0.01	min$^{-1}$	0.01	Alon (2006)
$\gamma _{S}$	Degradation rate of RsaL	0.0025	min$^{-1}$	0.01	Alon (2006)
$\gamma _{HL}$	Degradation rate of 3O-C12-HSL	0.01	min$^{-1}$	0.01	Alon (2006)
$\gamma _{RL}$	Degradation rate of LasR/3O-C12-HSL	0.01	min$^{-1}$	0.01	Alon (2006)
$\gamma _{mL}$	Degradation rate of lasI mRNA	0.2	min$^{-1}$	0.2	5 min half-life of RNA, Alon (2006)
$\gamma _{mS}$	Degradation rate of rsaL mRNA	0.2	min$^{-1}$	0.2	5 min half-life of RNA, Alon (2006)
$D _{HL}$	Diffusion constant of 3O-C12-HSL	60	min$^{-1}$	$0{-}10^{4}$	Pai and You (2009)
$H_\mathrm{ex}$	Concentration of extracellular 3O-C12-HSL	10	nM	10–100	Estimate
$R_{L0}$	Total concentration of LasR and LasR/3O-C12-HSL	200	nM	200	QseB in *E. coli*, Ishihama et al. (2014)

**Table 3:** Non-dimensional parameters involved in the model

Name	Description	Standard value	Range
$a_{1}$	The effect of extracellular signal molecules	0.01	$5\times 10^{-3}-10^{-1}$
$a_{2}$	A squared ratio of concentration of Las components	0.3	$25\times 10^{-6}-25$
$a_{3}$	The degradation of LasI relative to signal molecule production	0.3	$0-100$
$a_{4}$	The control of binding of RsaL to LasI	0.4	$0-25\times 10^{3}$
$a_{5}$	The degradation of RsaL relative to signal molecule production	0.075	$0-25$

Parameter $a_{2}$ depends on $K_{L}$ and represents the relative binding strength of the LasR dimer, and $a_{4}$ assigns binding control of RsaL to LasI through parameter $K_{SL}$; consequently, it gives the relative production of RsaL to LasI. Both parameters $a_{2}$ and $a_{4}$ have a wide potential range, $25\times 10^{-6}-25$ and $0-25\times 10^{3}$, respectively. This is because the affinity constant between transcriptional regulator protein to genes-mRNA is largely unmeasured and depends on the chain structure of the signal molecule (short or long chain; $1-1000$ nM) (Alon 2006).

Parameter $a_{3}$ is inversely proportional to $\beta _{HL}$, the HSL production rate. It is related to $a_{5}$, which describes the degradation of RsaL relative to HSL production. It is important to note that we set $a_5 < a_3$ due to the transcription factor being more stable than the synthase. We shall see that the parameters $a_3$ and $a_{1}$ allow for fold and Hopf bifurcations in the system, leading to bistability and oscillations, respectively, as well as excitable dynamics, and provide suitable control parameters that can be varied in experiments.

**Fig. 2:**
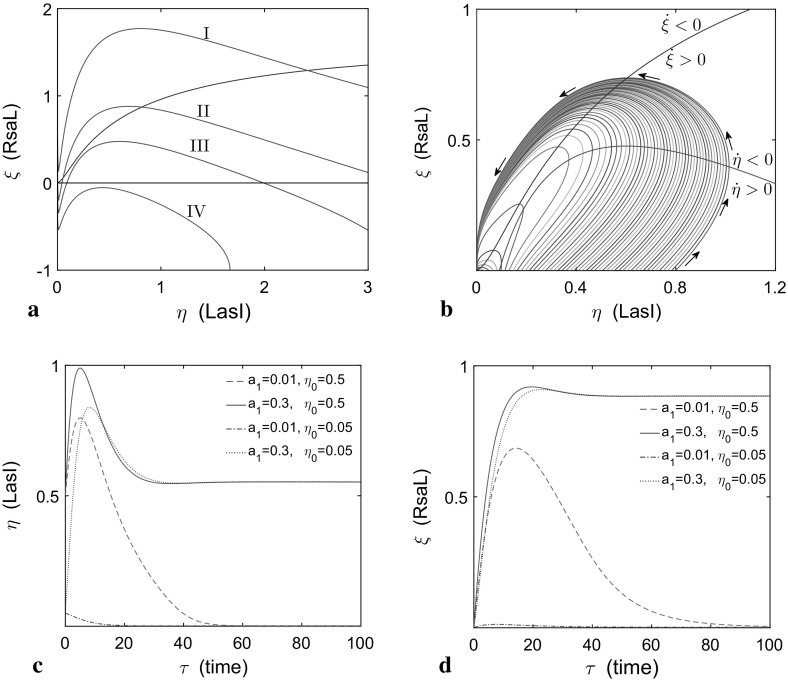
Qualitative dynamical behaviour of the *Pseudomonas aeruginosa* quorum sensing system. **a** Four phase portraits of interest resulting from intersection between LasI and RsaL ($\eta$ and $\xi$, respectively) nullclines. As the parameter $a_{3}$ varies, there can be one intersection point with no excitable dynamics (I and IV; for $a_{3}=0.1$ and 0.6, respectively), three intersection points (II; for $a_3 = 0.2$), or one intersection point with excitable dynamics (III; for $a_3 = 0.3$). **b** Excitable dynamics in the LasI and RsaL phase plane. Sufficiently large perturbations result in an excursion around the phase plane. **c**, **d** Time variation of $\eta$ and $\xi$ corresponding to the excitable trajectory of LasI and RsaL, respectively. All other parameters are as in Table 3 (Color figure online)

### Excitable Dynamics in the LasI and RsaL Phase Plane

The simplified system (15) and (16) involves just two variables, which we investigate with phase plane analysis. By varying $a_{3}$, four phase portraits of interest can be identified, as depicted in Fig. 2a. We plot the two nullclines $\dot{\eta }=0$ and $\dot{\xi }=0$, where $\eta$ and $\xi$ represent the dimensionless of LasI and RsaL concentration, respectively. Fixed points for the system lie at their intersection.

**Fig. 3:**
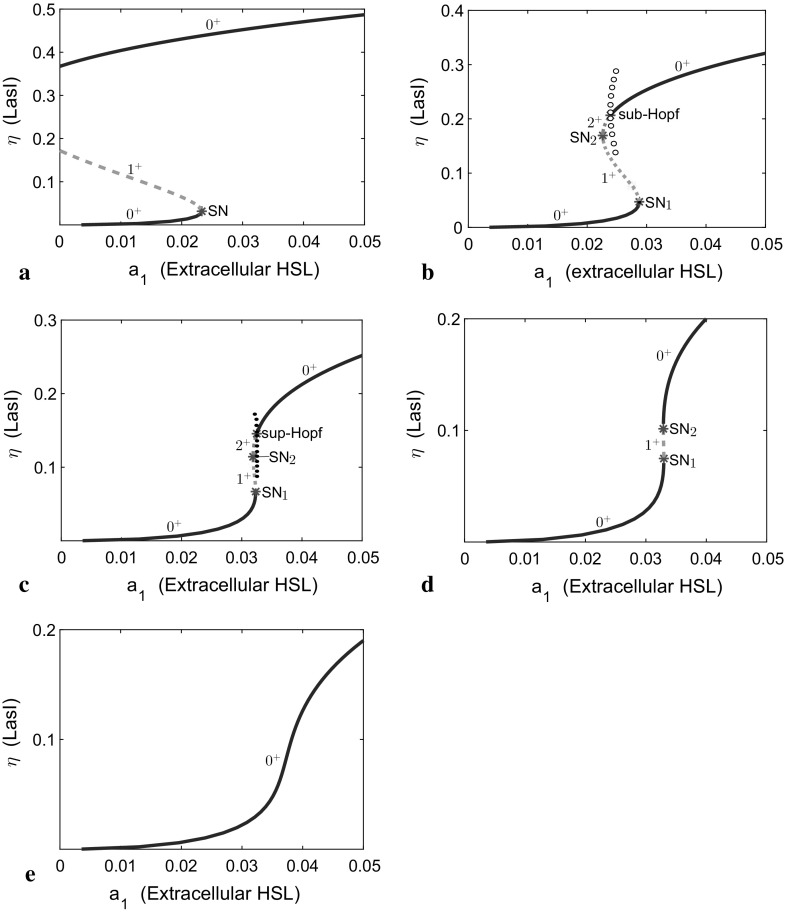
Bifurcation diagrams for autoregulation of the *las* system with respect to extracellular signal molecule concentration $a_1$ at five values of $a_3$: sub-figures **a**, **b**, **c**, **d**, and **e** are for $a_3 = 0.26, 0.3, 0.32, 0.323,$ and 0.34, respectively. *Solid lines* depict stable steady states (no eigenvalues with positive real part); *blue* and *green-dashed lines* depict unstable steady states with one or two eigenvalues with positive real part, *marked* $1^{+}$ or $2^{+}$, respectively; *open* (*solid*) *circles* denote maximum and minimum values of $\eta$(LasI) on unstable (stable) limit cycles. The *red stars* indicate values of parameter $a_1$ and coordinate $\eta$(LasI) at bifurcations. Co-dimension-1 singular points marked as SN indicate a saddle-node point (or limit point); *sub*-Hopf indicates a subcritical Hopf bifurcation point; *sup*-Hopf indicates a supercritical Hopf bifurcation point. All other parameters are as in Table 3 (Color figure online)

A qualitative description of the model behaviour is now possible, with reference to classical excitable systems such as the Fitzhugh–Nagumo model (Murray 1993). Cases I and IV (see Fig. 2a), which correspond to small and large values of the bifurcation parameter $a_3=0.1$ and 0.6, respectively, possess a single stable fixed point with the nullclines positioned so that there is no possibility of excitable behaviour (see below). For case II (Fig. 2a), there are two further intersections, which yield the central zone of the well-known *S*-shaped bifurcation diagram with three fixed points in a stable-unstable-stable configuration. This phenomenon has been reported in many experiments on autoregulation of genes (e.g. Alves and Dilo 2005; Angeli et al. 2004; Poignard 2014); more detail is presented in Fig. 3. The feedback loop that consists of both positive and negative autoregulation creates two possible LasI production states, “on” and “off” (at the large and small stable steady states, respectively), affecting signal molecule concentration. This is a familiar pattern that represents quorum sensing; the cells have a low steady state (off) from which it is possible to jump past an intermediate unstable state—either via stochastic fluctuations or changes in the external parameters, in particular the background level of HSL represented here by the parameter $a_1$—to a stable high steady state (on). Crucially, the return to the low steady state typically is not reversible and the system must trace a hysteretic loop to return to the low steady state via changes in the external parameters.

In Case III, there is a single fixed point and the nullcline $\dot{\eta }=0$ has a positive local maximum (which distinguishes it from cases II and IV); see Fig. 2a. This case allows for excitability in the LasI and RsaL phase plane caused by the $\dot{\eta }$ nullcline having cubic-like shape with a positive local maximum ($a_3 < 0.55$). By considering the behaviour of a perturbation to the steady-state solution (see Fig. 2b), if the production of the (*N*-(3-oxododecanoyl)-HSL) molecule is sufficiently small, then global stability of the single fixed point ensures that the trajectory returns to the steady state. The variable $\xi$ will engage, rapidly deplete and return the system to the small fixed point with a short loop. However, interesting behaviour occurs when there is a greater perturbation of (*N*-(3-oxododecanoyl)-HSL). For then the variable $\eta$ pushes the system to the right in the phase plane, so that there is an excitable pulse of (*N*-(3-oxododecanoyl)-HSL). Consequently, there is no short route back to the stable fixed point and the trajectory undergoes a large excursion before returning. A small region of parameter space in Case II can also result in excitability ($0.27 \le a_3 \le 0.28$). Here there are three intersections between $\eta$ and $\xi$ nullclines, which are low stable fixed point, an intermediate saddle and a high unstable fixed points. In this region of parameter space, the saddle-unstable fixed point region is not accessible to trajectories that initiate in a zone close to the stable fixed point (see “Appendix 2”, particularly Fig. 8).

The initial condition $\eta (0) = \eta _{0}$ and concentration of extracellular HSL have a large effect on the amplitude and duration of the excited pulse, as can be seen in Fig. 2. The results in Fig. 2b are plotted as a function of time in Fig. 2c, d, where the purple dashed line and red dashed line represent either excitable or non-excitable trajectories for large and small perturbations, respectively (for $a_1=0.01$ or, equivalently, $H_\mathrm{ex}=10 \mathrm{nM}$). By increasing external HSL concentration in the system via the variable $a_1$ (equivalent to increasing $H_\mathrm{ex}$ from 10 nM to $300\, \mathrm{nM}$), we find solutions tending to the single high steady state irrespective of the initial perturbation (shown in Fig. 2c, d by blue solid and black dotted lines; discussed later).

### Bifurcation Structure

The QS circuity of *P. aeruginosa* is complex, with the *las* system itself consisting of both positive and negative feedback loops. The *lasI* gene is activated by LasR/3O-C12-HSL leading to increased (*N*-(3-oxododecanoyl)-HSL) production, positive feedback. However, LasR/3O-C12-HSL also activates the *rsaL* gene and induces expression of RsaL. RsaL inhibits expression of *lasI* and *rsaL* genes, and this process constitutes negative feedback. In general, this negative feedback loop is employed to maintain a homeostatic balance in the system. However, the interaction of both positive and negative feedback can yield bistability (and associated *hysteresis*; Pfeuty and Kaneko 2009). There are many other studies on the dynamical behaviour of gene regulatory networks involving positive and negative feedback loops. Song et al. (2007) demonstrated that interlocked positive and negative feedback loops play essential roles in bistability and oscillations. More complex bifurcations of co-dimension one or two have also been explored (Hat et al. 2016). Varying $a_{1}>0$, the effect of extracellular signal molecule alters the qualitative dynamical behaviour of LasI and RsaL. Thus, initially we investigated the dynamics of our system with a one-parameter bifurcation analysis of LasI versus $a_{1}$. We used continuation methods to track the evolution of solutions for $\eta$ versus $a_{1}$.

**Fig. 4:**
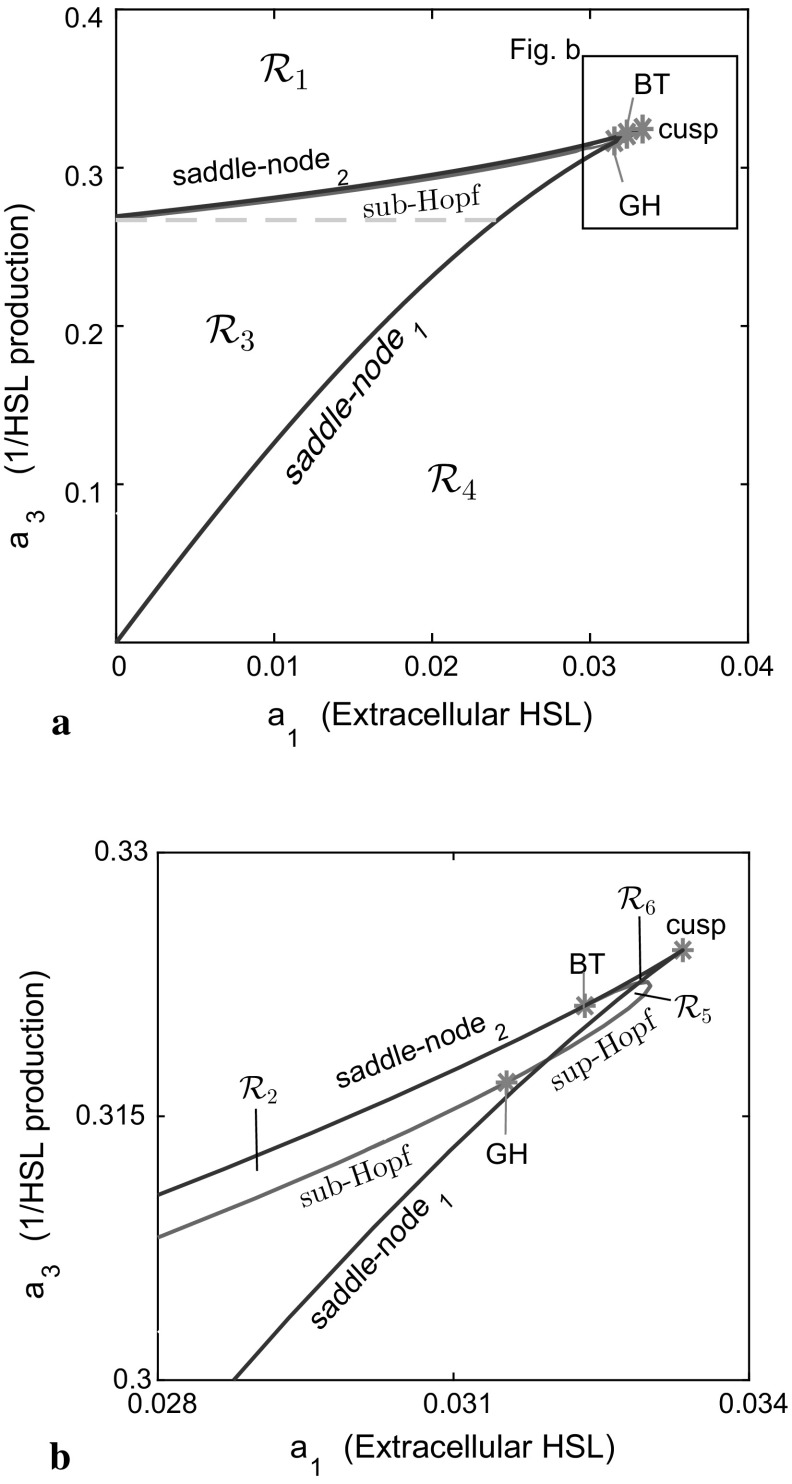
Two-dimensional bifurcation diagram for ($a_1,a_3$). The *bifurcation lines* divide the parameter domain into six regions $\mathcal{R}_1$,..., $\mathcal{R}_6$. Each of these regions is explained in the main text. The *blue lines* depict the *saddle-node lines*, defined by the locus of saddle-node bifurcation points (or limit points), with subscripts 1 or 2 as in Fig. (3). The Hopf lines are constructed by subcritical Hopf and supercritical Hopf bifurcation points, which are presented by *brown lines*. The bistable region, $\mathcal{R}_3$, consists of *reversible* (above of *grey-dash lines*) and *irreversible* (below of *grey-dash lines*). The *red stars* mark particular values of parameters $a_1$ and $a_3$ indicating a cusp, Bogdanov–Takens point (BT) or generalized-Hopf point (GH) (Color figure online)

Figure 3a–e depicts five qualitatively different dynamical behaviours, each at a different fixed value of $a_{3}$, the degradation of LasI relative to signal molecule production. As shown in Fig. 3a–d, there are at least four different bifurcation diagrams. Bifurcations of steady states do not appear in Fig. 3e. Figure 3a, for $a_3=0.26$, depicts the potential for *irreversible* bistability. The left-hand saddle-node bifurcation can cross the $\eta$-axis for sufficiently small $a_3$, preventing a drop from the upper to the lower branch. In addition, in Fig. 3b–d, *reversible* bistability of LasI is evident in the range of $a_1$ bounded by the two limit points, SN$_1$ and SN$_2$, which denote saddle-node bifurcations. In the low steady-state branch, the concentration of LasI is low until $a_1$ level exceeds the critical value SN$_{1}$ ($a_{1} = 0.029, 0.032,$ and 0.0329, for Fig. 3b–d, respectively), at which point the concentration of LasI increases abruptly to a high value. In similar manner, starting with $a_1$ high, the concentration of LasI does not drop significantly until $a_1$ reduces below a critical value, either SN$_{2}$ ($a_{1} = 0.023, 0.0318,$ and 0.0328, for Fig. 3b–d, respectively) or an earlier bifurcation such as a subcritical Hopf bifurcation. With $a_3=0.3$ (Fig. 3b), subcritical Hopf (sub-Hopf) bifurcation points exist for the high fixed point in the bistability region, at $a_{1} = 0.024$. The upper steady-state solution is unstable between SN$_2$ and sub-Hopf point and stable beyond the sub-Hopf point where $a_1>0.24$. For $a_3=0.32$ (Fig. 3c), the two saddle-nodes (SN$_{1}$ and SN$_{2}$) move close together. Consequently, the bistability region is narrow. Here, the Hopf bifurcation becomes supercritical (sup-Hopf; at $a_{1} = 0.032$). For $a_3=0.323$ (Fig. 3d), the sup-Hopf point disappears and the saddle-node bifurcations collide in Fig. 3e for $a_3\ge 0.325$; the bistable regime ceases to exist.

Figure 4 provides a two-dimensional bifurcation diagram in the ($a_1$, $a_3$)-plane. In general, for a value of $a_1$ smaller than at the cusp point, decreasing $a_3$ from a large value we move through a region with one relatively small stable fixed point for $\eta$ to one small stable fixed point with excitable dynamics. Decreasing $a_3$ further provides a bistable regime before eventually reaching a region with one large stable fixed point for sufficiently small $a_3$. The bistability region collapses through the collision of the two saddle-node lines at the cusp ($a_{1}=0.033$, $a_{3}=0.324$), a possibility that might be inferred from Fig. 2a. For $a_{1}>0.033$, the bistable regime ceases to exist.

Three co-dimension-2 singular points, a cusp point, a Bogdanov–Takens bifurcation point (BT), and a generalized-Hopf bifurcation point (GH), are identified in Fig. 4. The Bogdanov–Takens bifurcation, at $(a_{1},a_{3})=(0.032,0.321)$, is caused by coalescence of a saddle-node point (SN$_{2}$) and a Hopf bifurcation point (sup-Hopf). A sup-Hopf line extends to the lower right of the saddle-node lines close to the cusp in the bifurcation diagram. The generalized-Hopf bifurcation is at $(a_{1},a_{3})=(0.317,0.002)$, a transition point from sup-Hopf to sub-Hopf. It is clear that the bifurcation structure is complex, especially close to the cusp point, although the solution space is dominated by the fold bifurcation and excitability.

The response diagram (Fig. 4) illustrates that the bifurcation lines divide the $(a_{1},a_{3})$ parameter plane into six regions, as depicted by region $\mathcal{R}_{1}$,..., $\mathcal{R}_{6}$. Detailed phase diagrams associated with each regions are given in “Appendix 2”. In summary, $\mathcal{R}_{1}$ has either a monostable fixed point (one stable steady state) with low concentrations of LasI and RsaL for $a_{3} \ge 0.55$ (Fig. 8a), or excitable solutions, as discussed in the previous section (Fig. 8b). In the narrow region $\mathcal{R}_{2}$ (see Fig. 4b for detail figure), bounded by SN$_{2}$ and sub-Hopf, three steady states arise, but only the lower steady state is stable (Fig. 8c, d). Excitable solutions are also possible. Region $\mathcal{R}_{3}$ is divided into four domains with different behaviour. Three steady states arise in which the upper and lower steady states are stable, but the middle state is unstable. In the first domain, the upper state in $\mathcal{R}_{3}$ is surrounded by an unstable limit cycle (Fig. 8e). A homoclinic bifurcation arises to give the second domain (Fig. 8f). Upon decreasing $a_{3}$ further in this region, a high stable spiral exists with a large domain of attraction (Fig. 8g, h). Region $\mathcal{R}_{4}$ is monostable with high concentrations of LasI and RsaL (Fig. 8i). In the area approaching the cusp point $\mathcal{R}_{5}$ and $\mathcal{R}_{6}$, two small distinct regions arise (see Fig. 4b). Region $\mathcal{R}_{5}$ provides stable oscillations and $\mathcal{R}_6$ bistability. In summary, the bifurcation lines divide the (a$_{1}$, a$_{3}$)-plane into regions of distinct solution behaviour: monostability ($\mathcal{R}_{1}$ and $\mathcal{R}_{4}$), bistability or excitation ($\mathcal{R}_{2}$, $\mathcal{R}_{3}$, and $\mathcal{R}_{6}$), and oscillatory $\mathcal{R}_{5}$.

### Travelling Wave of a Pulse in a Linear Chain of Cells

We demonstrated the potential for the *las* system to act as a pulse generator for a single cell in the previous section. To illustrate how this effect may translate into cell–cell communication within a colony, we consider a simplified linear chain of cells. The goal is to provide proof of principle that it is possible to set up a pulse train when the individual cells are coupled to the dynamics of their neighbours. For simplicity, we assume that diffusion across each cell membrane is such that the intracellular concentration of HSL is at a kinetic equilibrium (i.e. $\frac{\mathrm{d}H_L}{\mathrm{d}t} = 0$, as before) and, additionally, assume that there is a neighbourhood surrounding each cell where the extracellular concentration can be modelled simply by

18 $$\begin{aligned} \frac{\mathrm{d}H_\mathrm{ex}}{\mathrm{d}t} = D_{HL} (H_L-H_\mathrm{ex}) - \varDelta H_\mathrm{ex}, \end{aligned}$$

where $\varDelta$ is the isotropic loss of $H_\mathrm{ex}$ from this loosely defined neighbourhood. Writing in a dimensionless form using Eqs. (11) and (14) we obtain

19 $$\begin{aligned} \frac{\mathrm{d}\zeta }{\mathrm{d}\tau } = \eta - D \zeta , \end{aligned}$$

where

20 $$\begin{aligned} \zeta = H_\mathrm{ex} \frac{\alpha _L \beta _L \beta _{HL}}{\gamma _{mL} D_{HL}^2 K_D^2} \text{, } \quad D = \varDelta \frac{\gamma _{mL} D_{HL} K_D}{\alpha _L \beta _L \beta _{HL}} \text{, } \quad K_D = \frac{k_L^-}{k_L^+}. \end{aligned}$$

Additionally, we construct a chain of otherwise identical systems, $(\eta _i,\zeta _i)$, for $i=1,2,\ldots$, coupled via the external concentrations of HSL in the local neighbourhood of each cell.

21 $$\begin{aligned} \frac{\mathrm{d}\zeta _{i}}{\mathrm{d}\tau } = \eta _i - D \left( (1 - 2d) \zeta _i + d \sum _{j = \pm 1} (\zeta _i - \zeta _{i+j})\right) , \end{aligned}$$

where $\zeta$ is the rescaled $H_\mathrm{ex}$ variable and *d* is now the fraction of the isotropic single loss that is retained in the single cell–cell interchange.

**Fig. 5:**
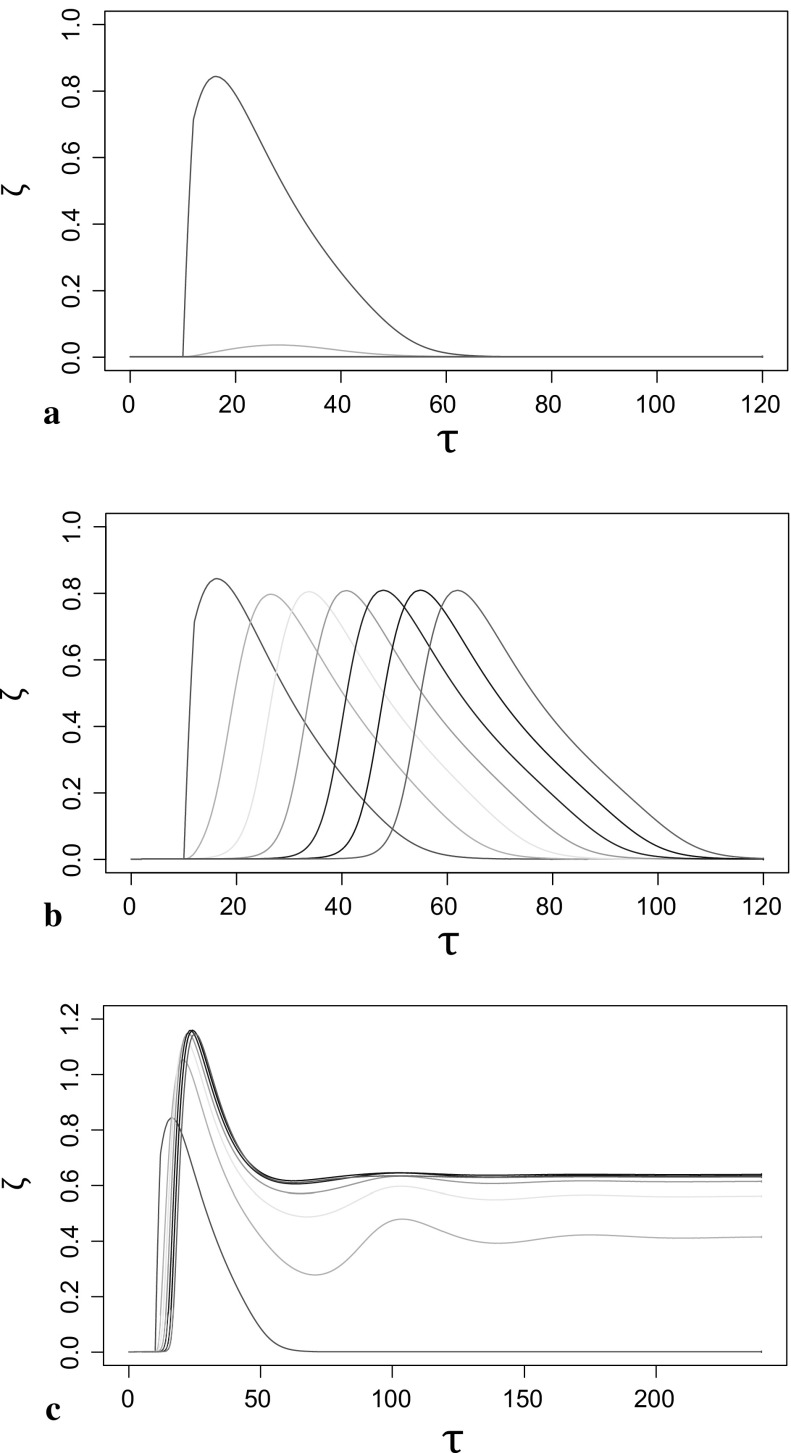
Pulse generation in the *las* system for single cells triggers a pulse train when the individual cells are coupled together. $x-$ and $y-$axes represent time variation of external HSL concentration ($\zeta$) corresponding to the pulse train, which consist of a linear chain of seven cells. Three types of solution are found when the coupling fraction *d* is varied: **a** $d=0.03$, **b** $d=0.1$, **c** $d=0.3$ (Color figure online)

**Fig. 8:**
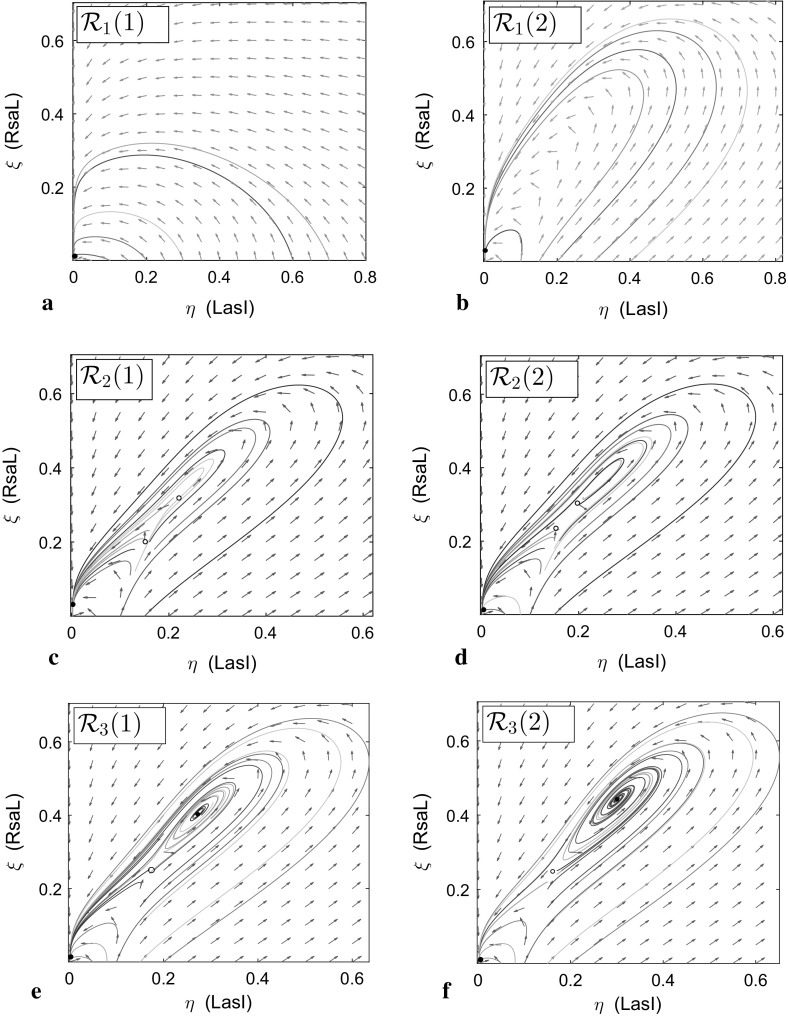
Phase diagram corresponding to the labelled regions in Fig. 4 (**a**, **b** in region $\mathcal{R}_{1}$; **c**, **d** in region $\mathcal{R}_{2}$; **e**–**h** in region $\mathcal{R}_{3}$; **i** in region $\mathcal{R}_{4}$; **j** in region $\mathcal{R}_{5}$). Stable (unstable) steady states are denoted by *solid* (*open*) *circles* (Color figure online)

In this simplified system, we can investigate whether it is possible for a single cell $i=1$ to propagate a signal. To do this, we trigger an arbitrary gain in the HSL, sufficient to trigger an excitable response from system $i=1$, and examine the downstream impact. To do this, we increase the levels of HSL by two orders of magnitude from the low steady state (QS off) for the equivalent lifetime of a single cell (corresponding to one dimensionless time unit) after the cells reach steady state. Three types of solution are presented in Fig. 5, illustrating a range of behaviour from no propagation of the signal for small coupling, propagation of a pulse for intermediate coupling, to propagation from a mutually supported high steady state (QS on) for large coupling.

## Discussion

We have described a model of the QS system in *P. aeruginosa* by considering nonlinear positive and negative feedback loops associated with the production of the synthase LasI and the regulator RsaL (Fagerlind et al. 2005; Pearson et al. 1997; Rampioni et al. 2007b). These nonlinear effects create the possibility of novel dynamical behaviours in the model. We have created a dimensionless set of equations to describe these behaviours and explored how the five dimensionless parameters affect the results whilst maintaining biological plausibility. Where possible we have taken parameters from the biological literature and this has led to significant deviation in our parameter choices from existing mathematical biology manuscripts, notably the work of Fagerlind et al. (2005); Fagerlind (2008), which needs commenting upon. When the parameters in these articles, which subsequently have been adopted in other later texts, were compared to biological estimates (presented for example in Alon (2006)), they were found to be significantly different. For example, the parameters used in (Fagerlind et al. 2005) suggest that the typical lifetime of a transcription factor is of the order of seconds, when biological estimates typically describe transcription factors as stable proteins with lifetimes of the same order as the cellular turnover time, i.e. hours for *Pseudomonas aeruginosa*. Furthermore, it suggests mRNA molecules are *more* stable than the proteins they are translated to, which is clearly at odds with biological knowledge about the bursty nature of protein production (Xie et al. 2008).

We are confident that our dimensionless parameters thus lie within a biologically plausible region, though we acknowledge that the effects we report here are sensitive to the values of these parameters. An additional complication is the possibility of other functional forms for the rate of transcription of mRNA from the *lasI* and *rsaL* genes as a function of the active forms of both LasR and RsaL. The form we present is one from a family of different choices that result in the same qualitative behaviour: our analysis, presented in the appendix, suggests that competitive binding between RsaL and LasR is a requirement for excitability but symmetrical effects, resulting in the negative action of RsaL on its own production, are not required (as is shown on many diagrams of the *las* system Dockery and Keener 2001). In the absence of specific biochemical data of this relationship at the intergenic region, our analysis represents a significant step forward in incorporating biochemical knowledge in mathematical models of quorum sensing. There are biological instances where the binding in the intergenic region decouples and the *rsaL* and *lasI* genes are promoted and repressed independently, notably in the genetic isolates obtained from cystic fibrosis patients (Rampioni et al. 2007b). In this case, the mathematical modelling of the system becomes trivial as the two systems are decoupled, and there is no possibility of excitation.

A survey of a realistic region of parameter space revealed a range of interesting numerical solution behaviour, such as limit cycles. The full equations, without approximations to reduce the number of equations, were also solved numerically and revealed similar solution behaviour (not shown). Continuation methods were employed for the reduced system in the $a_1$–$a_3$ plane to track bifurcations of the system of co-dimension one and two. The parameters $a_{1}$ and $a_{3}$ represent the information outside and inside the cell, respectively. We found fold and Hopf bifurcations, both of co-dimension one. Furthermore, there are Bogdanov–Takens and generalized-Hopf bifurcations, each of co-dimension two. For example, at a Bogdanov–Takens bifurcation, Hopf and fold curves in the plane intersect. However, most of the complex bifurcation structure is confined to a relatively small region of parameter space; the dominant behaviour is that of the fold bifurcation outside which are regions of excitable solutions.

We have demonstrated the potential for the *las* system to act as a pulse generator. As there is no explicit feedback from *rhl* to *las*, we may consider the *las* system as a black-box controller for the *rhl* system. We have demonstrated that this can lead to the generation of a pulse train when the individual cells are coupled by diffusion of the HSL signal molecule. These observations allow for a novel stigmergy (Gloag et al. 2015) in the coupled *las/rhl* system; that of a *quorum memory*. The *las* system is coupled to the *rhl* system in two distinct ways; the active LasR transcription factor promotes the transcription of its counterpart in the *rhl* system, RhlR, and due to competitive binding on the RhlR molecule of the two homoserine lactones the presence of 3-oxo-C12-HSL acts to prevent the activation of RhlR by C4-HSL. This creates an interesting unreported effect in the combined system: what one might call a ‘handbraked acceleration’. Assuming the *rhl* system follows the same Lux-like dynamics of the *las* system, the additional production of RhlR increases the likelihood of the system switching to its higher steady state, but this is tempered, or handbraked, by the presence of significant amounts of 3-oxo-C12-HSL preventing the activation of the rhlR system and its downstream consequences, notably rhamnolipid production. However, the effect of the pulses (the ‘revs’ of the ‘acceleration’) is still to increase the amount of RhlR, sustained due to its relative stability compared to the diffusing homoserine lactones, so that when the handbrake of 3-oxo-C12-HSL is removed the system has an increased likelihood of activating the *rhl* system. In effect the cells have been primed by the pulses and thus have a memory of experiencing higher densities of cells (or low diffusion regions Redfield 2002). Cells will lose memory only when they no longer experience a local environment rich in the 3-oxo-C12-HSL from the *las* system. Therefore, the competition between the two signal molecules, one produced by the *las* system and the other produced by the *rhl* system, may enable cells to trigger rhamnolipid production only when they are at the edge of an established aggregation. This suggests a previously unreported reason for the coupled nature of the *las* and *rhl* systems and a mechanism for how they act in tandem to create a sophisticated control system for sociality and virulence in this important pathogenic organism.

## Figures and Tables

**Fig. 6:**
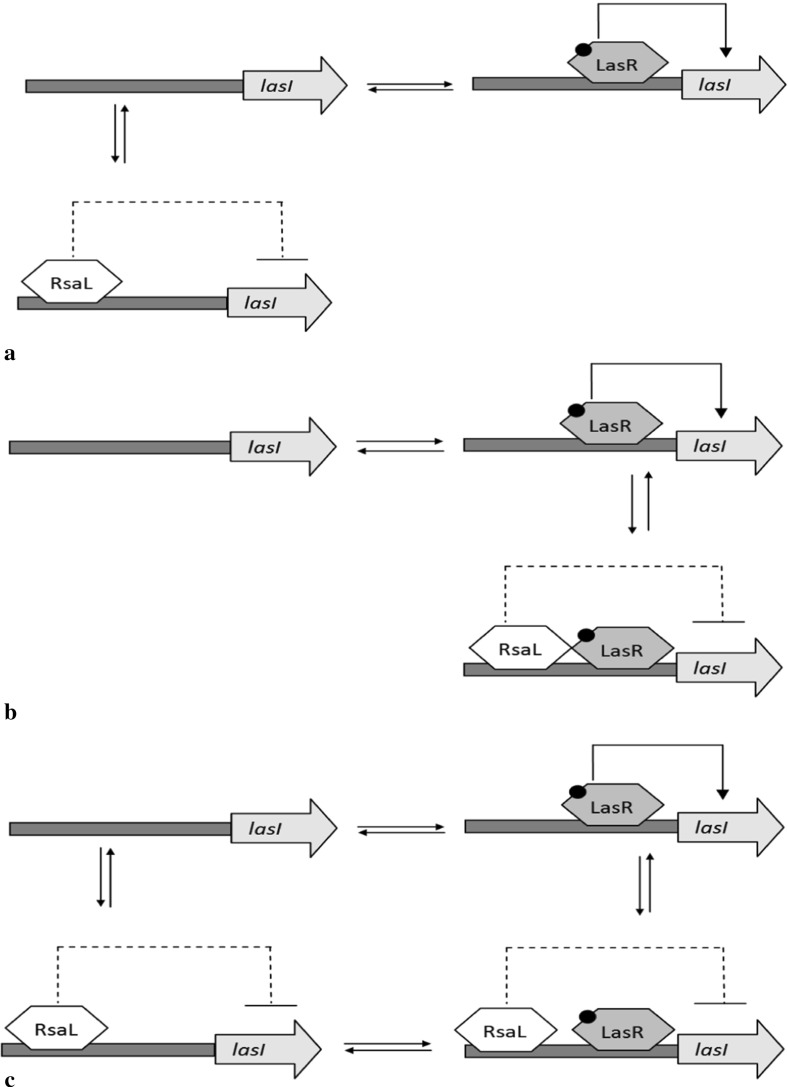
Binding types of inhibition: **a** competitive, **b** uncompetitive, and **c** non-competitive inhibition (Color figure online)

**Fig. 7:**
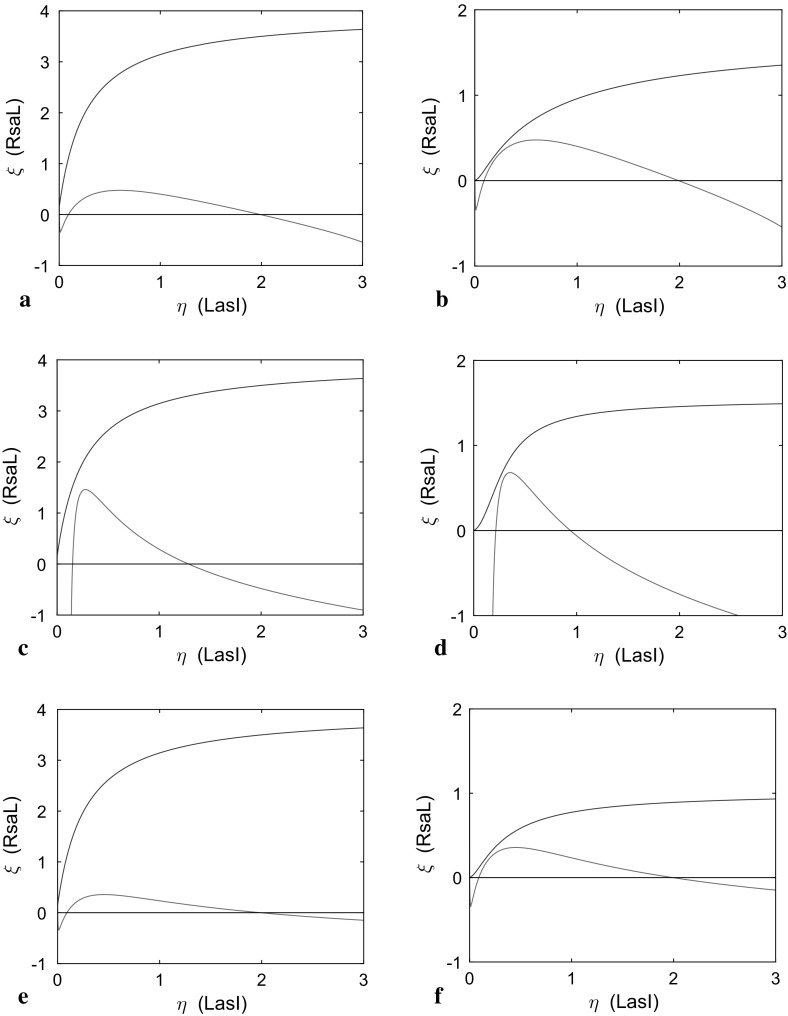
Intersection between LasI and RsaL concentration nullclines with different types of inhibition: **a** non-symmetric competitive, **b** symmetric competitive, **c** non-symmetric uncompetitive, **d** symmetric uncompetitive, **e** non-symmetric non-competitive, and **f** symmetric non-competitive (Color figure online)

**Fig. 9:**
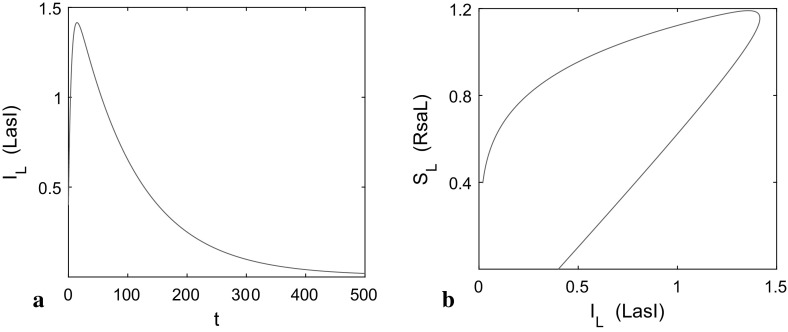
**a** Time-dependent solutions from the full system of equations before systematic reduction, corresponding to the excitable trajectory of LasI. **b** Excitable dynamics in the LasI-RsaL phase plane (Color figure online)

## Appendix 1

RsaL is thought to work as an inhibitor in the *las* system on *P. aeruginosa* and, therefore, the Michaelis–Menten equation can be applied. Whilst there are a considerable number of studies about the QS system for *P. aeruginosa*, to the best knowledge of the authors, none of these studies provide sufficient biochemical evidence to determine whether the binding of RsaL in the intergenic region of the *las* system is competitive, uncompetitive, non-competitive, or mixed-competitive inhibition (using the standard biochemical descriptors). Here, we restrict attention to competitive, uncompetitive and non-competitive inhibition (mixed-competitive inhibition represents a combination of both competitive and uncompetitive inhibition, such that the RsaL inhibitor binds LasR/3O-C12-HSL or LasR/3O-C12-HSL+*lasI* genes with different affinities; mixed-competitive is the same as non-competitive inhibition if genes are bound with equal affinity). Furthermore, there is insufficient biochemical evidence whether the expression rates for *lasI* and *rsaL* genes are symmetric or not (Fig. 6).

### Competitive Inhibition

Competitive binding occurs when both the LasR/3O-C12-HSL enzyme and RsaL inhibitor compete for binding to the active sites of the *lasI* gene (see Fig. 6a). Thus, the governing equations result from a blockade of either positive or negative *lasI* gene interaction by either LasR/3O-C12-HSL or RsaL, respectively. Furthermore, this competitive inhibition can be classified into non-symmetric and symmetric binding based on expression rates for LasR/3O-C12-HSL to *lasI* and *rsaL* genes.

Due to the overlapping of binding sites, LasI is completely inactive when the RsaL inhibitor binds *lasI* genes. For the non-symmetric case, the affinity constant between LasR/3O-C12-HSL and *rsaL* is relatively small. Meanwhile, in the symmetric case the affinity constant between LasR/3O-C12-HSL and *rsaL* gene is identical to the affinity constant between LasR/3O-C12-HSL and the *lasI* gene.

#### Non-symmetric Competitive Inhibition

Here, the governing equations are

22 $$\begin{aligned} \frac{\mathrm{d}\hat{I}_{L}}{\mathrm{d}t}= & {} \beta _{L}\frac{R_{LH}^{2}}{K_{L}^{2}(1+S_{L}/K_{SL})^{2} +R_{LH}^{2}}-\gamma _{mL}\hat{I}_{L}+\beta _{L0}, \end{aligned}$$

23 $$\begin{aligned} \frac{\mathrm{d}\hat{S}_{L}}{\mathrm{d}t}= & {} \beta _{S}\frac{R_{LH}}{K_{S}+R_{LH}}-\gamma _{mS}\hat{S}_{L} +\beta _{S0}. \end{aligned}$$

Therefore, the non-dimensional model becomes

24 $$\begin{aligned} \frac{\mathrm{d}\eta }{\mathrm{d}\tau }= & {} \frac{\left( \eta +a_{1}\right) ^{2}}{a_{2}\left( 1+\xi \right) ^{2}\left( \eta +a_{1}+1\right) ^{2}+\left( \eta +a_{1}\right) ^{2}}-a_{3}\eta , \end{aligned}$$

25 $$\begin{aligned} \frac{\mathrm{d}\xi }{\mathrm{d}\tau }= & {} \frac{a_{4}\left( \eta +a_{1}\right) }{a_{6}\left( \eta +a_{1}+1\right) +\left( \eta +a_{1}\right) }-a_{5}\xi . \end{aligned}$$

#### Symmetric Competitive Inhibition

In this case, we find that

26 $$\begin{aligned} \frac{\mathrm{d}\hat{I}_{L}}{\mathrm{d}t}= & {} \beta _{L}\frac{R_{LH}^{2}}{K_{L}^{2}(1+S_{L}/K_{SL})^{2} +R_{LH}^{2}}-\gamma _{mL}\hat{I}_{L}+\beta _{L0}, \end{aligned}$$

27 $$\begin{aligned} \frac{\mathrm{d}\hat{S}_{L}}{\mathrm{d}t}= & {} \beta _{S}\frac{R_{LH}^{2}}{K_{L}^{2}(1+S_{L}/K_{SL})^{2} +R_{LH}^{2}}-\gamma _{mS}\hat{S}_{L}+\beta _{S0}, \end{aligned}$$

yielding the non-dimensional model

28 $$\begin{aligned} \frac{\mathrm{d}\eta }{\mathrm{d}\tau }= & {} \frac{\left( \eta +a_{1}\right) ^{2}}{a_{2}\left( 1+\xi \right) ^{2}\left( \eta +a_{1}+1\right) ^{2}+\left( \eta +a_{1}\right) ^{2}}-a_{3}\eta , \end{aligned}$$

29 $$\begin{aligned} \frac{\mathrm{d}\xi }{\mathrm{d}\tau }= & {} \frac{a_{4}\left( \eta +a_{1}\right) ^{2}}{a_{2}\left( 1+\xi \right) ^{2}\left( \eta +a_{1}+1\right) ^{2}+\left( \eta +a_{1}\right) ^{2}}-a_{5}\xi . \end{aligned}$$

### Uncompetitive Inhibition

Uncompetitive binding occurs when the RsaL inhibitor binds to a site which only becomes available after the LasR/3O-C12-HSL enzyme has bound to the active site of the *lasI* gene (see Fig. 6b). It can also be classified into non-symmetric and symmetric binding types, as indicated below.

#### Non-symmetric Uncompetitive Inhibition

Here,

30 $$\begin{aligned} \frac{\mathrm{d}\hat{I}_{L}}{\mathrm{d}t}= & {} \beta _{L}\frac{R_{LH}^{2}}{K_{L}^{2}+R_{LH}^{2}(1+S_{L}/K_{SL}^{'})^{2}}-\gamma _{mL}\hat{I}_{L}+\beta _{L0}, \end{aligned}$$

31 $$\begin{aligned} \frac{\mathrm{d}\hat{S}_{L}}{\mathrm{d}t}= & {} \beta _{S}\frac{R_{LH}}{K_{S}+R_{LH}}-\gamma _{mS}\hat{S}_{L} +\beta _{S0}, \end{aligned}$$

so that the non-dimensional model becomes

32 $$\begin{aligned} \frac{\mathrm{d}\eta }{\mathrm{d}\tau }= & {} \frac{\left( \eta +a_{1}\right) ^{2}}{a_{2}\left( \eta +a_{1}+1\right) ^{2}+\left( \eta +a_{1}\right) ^{2}\left( 1+\xi '\right) ^{2}}-a_{3}\eta , \end{aligned}$$

33 $$\begin{aligned} \frac{\mathrm{d}\xi }{\mathrm{d}\tau }= & {} \frac{a_{4}\left( \eta +a_{1}\right) }{a_{6}\left( \eta +a_{1}+1\right) +\left( \eta +a_{1}\right) }-a_{5}\xi , \end{aligned}$$

where $\xi '$ is a rescaled $\xi$.

#### Symmetric Uncompetitive Inhibition

For this case,

34 $$\begin{aligned} \frac{d\hat{I}_{L}}{\mathrm{d}t}= & {} \beta _{L}\frac{R_{LH}^{2}}{K_{L}^{2}+R_{LH}^{2} (1+S_{L}/K_{SL}^{'})^{2}}-\gamma _{mL}\hat{I}_{L}+\beta _{L0}, \end{aligned}$$

35 $$\begin{aligned} \frac{\mathrm{d}\hat{S}_{L}}{\mathrm{d}t}= & {} \beta _{S}\frac{R_{LH}^{2}}{K_{L}^{2}+R_{LH} (1+S_{L}/K_{SL}^{'})^{2}}-\gamma _{mS}\hat{S}_{L}+\beta _{S0}, \end{aligned}$$

such that the non-dimensional model is

36 $$\begin{aligned} \frac{\mathrm{d}\eta }{\mathrm{d}\tau }= & {} \frac{\left( \eta +a_{1}\right) ^{2}}{a_{2}\left( \eta +a_{1}+1\right) ^{2}+\left( \eta +a_{1}\right) ^{2}\left( 1+\xi '\right) ^{2}}-a_{3}\eta , \end{aligned}$$

37 $$\begin{aligned} \frac{\mathrm{d}\xi }{\mathrm{d}\tau }= & {} \frac{a_{4}\left( \eta +a_{1}\right) ^{2}}{a_{2}\left( \eta +a_{1}+1\right) ^{2}+\left( \eta +a_{1}\right) ^{2}\left( 1+\xi '\right) ^{2}}-a_{5}\xi . \end{aligned}$$

Here, $K_{SL}^{'}$ represents the dissociation constant for the RsaL inhibition of binding of *lasI* genes to the LasR/3O-C12-HSL enzyme. It is different to $K_{SL}$, which represents the dissociation constant of inhibitor RsaL on *lasI* genes.

### Non-competitive Inhibition

Non-competitive inhibition occurs when the RsaL inhibitor can bind to the free *lasI* gene or the *lasI*-bound LasR/3O-C12-HSL enzyme (see Fig. 6c). As explained above, the non-competitive inhibition form is a special case of mixed-competitive inhibition, where it binds to the target with two equal inhibition constants.

#### Non-symmetric Non-competitive Inhibition

Here,

38 $$\begin{aligned} \frac{\mathrm{d}\hat{I}_{L}}{\mathrm{d}t}= & {} \beta _{L}\frac{R_{LH}^{2}}{K_{L}^{2}+R_{LH}^{2}}\frac{1}{(1+S_{L}/K_{SL})^{2}}-\gamma _{mL}\hat{I}_{L}+\beta _{L0}, \end{aligned}$$

39 $$\begin{aligned} \frac{\mathrm{d}\hat{S}_{L}}{\mathrm{d}t}= & {} \beta _{S}\frac{R_{LH}}{K_{S}+R_{LH}}-\gamma _{mS}\hat{S}_{L} +\beta _{S0}, \end{aligned}$$

providing the non-dimensional model

40 $$\begin{aligned} \frac{\mathrm{d}\eta }{\mathrm{d}\tau }= & {} \frac{\left( \eta +a_{1}\right) ^{2}}{a_{2}\left( \eta +a_{1}+1\right) ^{2}+\left( \eta +a_{1}\right) ^{2}}\frac{1}{\left( 1+\xi \right) ^{2}}-a_{3}\eta , \end{aligned}$$

41 $$\begin{aligned} \frac{\mathrm{d}\xi }{\mathrm{d}\tau }= & {} \frac{a_{4}\left( \eta +a_{1}\right) }{a_{6}\left( \eta +a_{1}+1\right) +\left( \eta +a_{1}\right) }-a_{5}\xi . \end{aligned}$$

#### Symmetric Non-competitive Inhibition

Here, the governing equations are

42 $$\begin{aligned} \frac{\mathrm{d}\hat{I}_{L}}{\mathrm{d}t}= & {} \beta _{L}\frac{R_{LH}}{K_{L}+R_{LH}}\frac{1}{(1+S_{L}/K_{SL})}-\gamma _{mL}\hat{I}_{L}+\beta _{L0}, \end{aligned}$$

43 $$\begin{aligned} \frac{\mathrm{d}\hat{S}_{L}}{\mathrm{d}t}= & {} \beta _{S}\frac{R_{LH}}{K_{L}+R_{LH}}\frac{1}{(1+S_{L}/K_{SL})}-\gamma _{mS}\hat{S}_{L}+\beta _{S0}, \end{aligned}$$

and the non-dimensional model is

44 $$\begin{aligned} \frac{\mathrm{d}\eta }{\mathrm{d}\tau }= & {} \frac{\left( \eta +a_{1}\right) ^{2}}{a_{2}\left( \eta +a_{1}+1\right) ^{2}+\left( \eta +a_{1}\right) ^{2}}\frac{1}{\left( 1+\xi \right) ^{2}}-a_{3}\eta , \end{aligned}$$

45 $$\begin{aligned} \frac{\mathrm{d}\xi }{\mathrm{d}\tau }= & {} \frac{a_{4}\left( \eta +a_{1}\right) ^{2}}{a_{2}\left( \eta +a_{1}+1\right) ^{2}+\left( \eta +a_{1}\right) ^{2}}\frac{1}{\left( 1+\xi \right) ^{2}}-a_{5}\xi . \end{aligned}$$

Using the simplification steps highlighted in the main text, we determine appropriate nullclines for each binding type (see Fig. 7). The equations resulting from non-symmetric and symmetric competitive behaviour are presented in Fig. 7a, b, revealing the same qualitative behaviour as for the system in the main text. This suggests that competitive binding between RsaL and LasR/3O-C12-HSL with or without symmetry is sufficient for excitable behaviour. Therefore, although (Rampioni et al. 2007b) suggest there may be negative feedback from RsaL to its own production, this symmetrical binding is not required for excitable behaviour (c.f. general diagrams of the *las* system in Van Delden and Iglewski 1998; De Kievit et al. 2002; Dockery and Keener 2001; Fagerlind et al. 2005; Schaadt et al. 2013).

## Appendix 2

Further investigation of the structure in Fig. 4 finds that the $(a_{1},a_{3})$ parameter plane is divided by bifurcation lines into six regions. The six corresponding phase diagrams of the dynamic solution behaviour for LasI and RsaL are illustrated below. In order to get clear understanding of qualitative behaviour for region $\mathcal{R}_{1}$ to $\mathcal{R}_{4}$, we fix parameter values for $a_{1}, a_{2}, a_{4}, a_{5}$ (see Table 3) and vary $a_{3}$ from a high to a low value. For Fig. 8a–i, $a_{3} = 0.6, 0.3, 0.281, 0.28, 0.278, 0.275, 0.27, 0.2,$ and 0.1, respectively.

In the region $\mathcal{R}_{1}$, the system has a single low stable steady state, i.e. with 0 eigenvalues with positive real part. In this region, solutions have two qualitatively different behaviours (see Fig. 8a, b). The difference between those two behaviours is that the large perturbation in Fig. 8b has a large excursion around the phase plane before it reaches the stable fixed point. In the region $\mathcal{R}_{2}$ after a saddle-node bifurcation, system has 0, 1, and 2 eigenvalues with positive real part in the low, middle and high steady states, respectively. Thus, it consists of one stable low fixed point, one saddle and one unstable fixed point. There is a change from unstable fixed point to unstable spiral (see Fig. 8c, d).

In the region $\mathcal{R}_{3}$ after a subcritical Hopf bifurcation, the system has 0, 1, and 0 eigenvalues with positive real part in the low, middle and high states, respectively. In this region, the system has four different qualitative behaviours. Upon decreasing $a_{3}$, the journey of qualitative dynamical behaviour can be seen clearly in Fig 8e–h. First, the unstable limit cycle coexists with one saddle and two stable steady states (see Fig. 8e). Then, a homoclinic bifurcation occurs (a collision between the unstable limit cycle and the saddle; see Fig. 8e) resulting in a high state stable spiral with large basin of attraction (see Fig. 8f). Lastly, the saddle-node moves closer to the low stable fixed point (see Fig. 8h) eventually colliding via a saddle-node bifurcation to yield region $\mathcal{R}_{4}$. This system has one high state fixed point with 0 eigenvalues with positive real part (see Fig. 8i).

In Fig. 4, we find two other regions, $\mathcal{R}_{5}$ and $\mathcal{R}_{6}$. In region $\mathcal{R}_{5}$, a stable limit cycle and one unstable steady state exists (see Fig. 8j, $a_1 = 0.03298, a_2 = 0.3, a_3 = 0.3226, a_4 = 0.4$ and $a_5 = 0.075$). In region $\mathcal{R}_{6}$, the system has one unstable and two stable fixed points, but the region is very small as it is close to the cusp where the two saddle-node bifurcations collide.

## Appendix 3

In this paper, the reduced model of the QS system for *P. aeruginosa* explored in the main text reveals the potential for excitable pulse generation in the *las* subsystem. However, numerical solution of the full system of seven differential equations reveals very similar solution behaviour, including excitable pulse generation. Excitation occurs when there is a sufficiently large perturbation of (*N*-(3-oxododecanoyl)-HSL) leading to pulse production of LasI, pushing the system to the right in the LasI-RsaL projected phase plane. Typically, this is followed by a large excursion around phase space before returning to the stable fixed point (see Fig. 9a, b). Other solutions are also possible analogous to those in the reduced system. These results provide confidence that the behaviour of the full system has been adequately captured by the reduced model.
